# Efficacy of pain management strategies in adults with Amyotrophic Lateral Sclerosis (ALS): A Systematic Review

**DOI:** 10.1007/s10072-024-07643-0

**Published:** 2024-07-05

**Authors:** Juan Camilo Rojas-López, Pablo Isaac Estrada-Gualdron, Sofía Ramírez-Guerrero, Maria J. Velásquez-Cárdenas, Jesús Redondo-Escobar, Sofía Vargas-Arenas, Leonardo Palacios-Sánchez, Ximena Palacios-Espinosa

**Affiliations:** 1 Neuroscience Research Group (Neuros), Center of Neuroscience - Neurovitae, School of Medicine and Health Sciences, Universidad del Rosario, Bogotá, Colombia; 2 Grupo de Investigación Individuo, Familia y Sociedad Psychology Program, School of Medicine and Health Sciences, Universidad del Rosario, Bogotá, Colombia; 3 Neuroscience Research Seedbed (Semineuros), Center of Neuroscience - Neurovitae, School of Medicine and Health Sciences, Universidad del Rosario, Bogotá, Colombia

**Keywords:** Amyotrophic lateral sclerosis, Pain, Pain management, Medication therapy management

## Abstract

Amyotrophic lateral sclerosis (ALS) is a neurodegenerative disease characterized by progressive muscle weakness. Presence of pain in ALS patients is heterogeneously reported in studies, and mostly underrepresented in symptom scales. The aim of this study is to evaluate the efficacy of pharmacological and non-pharmacological therapeutic modalities for pain management in patients with ALS. A systematic review was conducted in four databases; PubMed, Scopus, Clinicaltrials.gov, and Cochrane-Ovid. Five randomized controlled clinical trials were included regarding pharmacological and non-pharmacological pain management interventions in adult patients with confirmed diagnosis of ALS in whom pain was objectively evaluated. Risk of bias assessment was evaluated using the RoB2.0 tool. Eligible studies were reported as a descriptive analysis. This systematic review was registered with PROSPERO ID: CRD42024495009. Five clinical trials regarding pain management strategies in ALS were eligible for analysis. Two out of five were non-pharmacological approaches whilst the remaining three provided pharmacological therapies. Of these, Mexiletine was efficient in terms of pain relief, particularly between 600 and 900 mg per day, whereas Mecasin showed no pain relief at both, high and low doses. Non-pharmacological therapies, such as exercise and osteopathic manual treatment also lacked efficacy in regard to pain management. Clinical trials focusing on pain management strategies for ALS patients are limited. Medical professionals, understandably focused on immediate life-threatening aspects, may inadvertently sideline the nuanced and intricate dimension of pain experienced by patients with ALS.

**Supplementary Information:**

The online version contains supplementary material available at 10.1007/s10072-024-07643-0.

## Introduction

Amyotrophic lateral sclerosis (ALS) is a neurodegenerative disease characterized by progressive muscle weakness, distinguished by damage to the upper and lower motor neuron [1–3]. It is influenced by a complex interaction between genetic and environmental factors leading to the dysfunction of neurons in the brain and spinal cord [1, 4, 5]. Risk of developing ALS increases progressively up to the eighth decade, with an average age of onset of 63 years [6, 7] and an incidence of 1.75–3 cases per 100,000 people per year [8, 9]. In terms of prevalence, Europe reported 10–12 cases per 100,000 people [10], followed by the United States with 11.80 per 100,000 people [11]. Nonetheless, epidemiological data in ALS is scarce and variables across countries [11, 12]. This condition has a marked predominance in men, with a risk of sporadic ALS of 1:350 compared to 1:400 in women [13]. Familial ALS represents 10–15% of cases [14], for instance, male carriers of C9ORF72 have a higher tendency to develop ALS at an earlier age than female carriers [1].

Although the focus on preserving basic functions like eating and breathing is paramount, an aspect often overshadowed is the profound impact of pain in the lives of ALS patients [15]. Pain is an unpleasant sensory and emotional experience associated with actual or potential tissue damage that has been crucial to delineate better therapeutic strategies for this disease [2, 15]. Some studies report the presence of pain in up to 85% of ALS patients, while others indicate a lower prevalence of around 15% [2, 3, 16]. This variation could be attributed to heterogeneous ways in which this symptom is assessed. Furthermore, neuropathic pain as one of the primary causes of pain can develop due to damage to somatosensory pathways [15, 17]. As for secondary causes, nociceptive pain resulting from tissue damage has also been studied. Among the painful symptoms, the most described are burning, tingling, allodynia, hyperalgesia, cramps and spasticity, among others [15, 17]. The most common reported locations of pain in patients with ALS are the back (50%), followed by the limbs (47%) and joints (42%) [2].

Over recent decades, peripheral sensory abnormalities, including the evidence of cutaneous denervation, have been reported among the non-motor manifestations in ALS [18, 19]. Increasing evidence suggests that ALS is a multisystem neurodegenerative disorder, also considered as a small fiber neuropathy (SFN) as recognized by skin biopsy studies in distal legs, irrespective of the disease duration [20]. Intraepidermal nerve fiber loss is a feature of most ALS patients. However, a correlation between cutaneous innervation and clinical features as onset, phenotype, course, and severity of the disease has not been found. A recent study assesses sensory involvement by applying a morpho-functional approach to a large population of ALS patients stratified according to King's stages and correlates these findings with the severity and prognosis of the disease. The study shows that in patients with ALS, peripheral sensory involvement worsens in parallel with motor disability. Furthermore, the correlation between skin innervation and disease activity may suggest the use of skin innervation as a potential prognostic biomarker [18, 20].

Pharmacological treatments are the most common therapeutic strategy used for pain management in ALS, with different medications administered depending on the type of pain the patient is experiencing. The most frequently administered drugs are gabapentin [21–24], pregabalin [15], tricyclic antidepressants [21, 25, 26], quinine sulphate [21, 27, 28], mexiletine [29], dronabinol [30, 31], cannabis [30, 32, 33], levetiracetam [34], NSAIDs [35, 36], opioids [35, 37], and baclofen [21, 38], among others. On the other hand, non-pharmacological treatments have been used, such as daily stretching [39], moderate physical activity [39], exercise [40], massages [41], acupuncture [42], and osteopathy [43].

A prior review in 2013 [35], was marked by a scarcity of studies and an absence of Randomized Controlled Trials (RCTs) on the matter, underscoring the critical need for a fresh perspective. Similarly, the latest systematic review on pain management strategies in patients with ALS published in 2017, found no conclusive evidence from RCTs [44]. Nonetheless, it provided insights into cramp treatment, indicating memantine and tetrahydrocannabinol (THC) may be ineffective, while vitamin E could offer a limited relief. In response, our review strategically narrows its focus to RCTs, ensuring a rigorous and evidence-based evaluation of therapeutic interventions for pain in ALS.

ALS causes a negative impact on the patient's life, leading to a change in routine and a progressive loss of autonomy [45]. This decline in autonomy results in a greater need for assistance in daily living activities, restructuring in eating habits, difficulties in communication, and impairment of the emotional state [45].

The aim of this study is to evaluate the efficacy of pharmacologic and non-pharmacological therapeutic modalities in terms of pain management and quality of life improvement in ALS. The prevailing attitude towards ALS often centers around the urgency of maintaining basic life functions, such as the ability to eat and breathe. However, this perspective, while crucial, sometimes results in the inadvertent neglect of the pain that accompanies the disease. This review seeks to challenge this status quo, advocating for a more holistic approach that recognizes and addresses the multifaceted nature of ALS, where pain management is as pivotal as other life-sustaining measures.

## Methods

This systematic review was registered in the International prospective register of systematic reviews (PROSPERO ID: CRD42024495009). This study was performed according to the Preferred Reporting Items for Systematic Reviews and Meta-Analyses (PRISMA) guidelines (Supplementary material 1).

### Data search strategy

The following databases were consulted to identify eligible studies; PubMed, Clinicaltrials.gov, Cochrane -Ovid and Scopus. The search was conducted on November 23rd, 2023 for all four databases. Full search strategies for all databases are presented below (Table 1). Duplicates were identified and removed using Zotero.

**Table 1:** Data search strategy per database

Database	Search strategy	Number of results
PubMed	((("pain"[MeSH Terms] OR "pain"[All Fields] OR "pain"[MeSH Terms] OR "pain"[Title/Abstract] OR "pain management"[Title/Abstract] OR "pain management"[MeSH Terms] OR ("pain management"[MeSH Terms] OR ("pain"[All Fields] AND "management"[All Fields]) OR "pain management"[All Fields])) AND "ALS"[All Fields]) OR ("amyotrophic lateral sclerosis"[MeSH Terms] OR ("Amyotrophic"[All Fields] AND "Lateral"[All Fields] AND "Sclerosis"[All Fields]) OR "amyotrophic lateral sclerosis"[All Fields]) OR "amyotrophic lateral sclerosis"[MeSH Terms] OR "amyotrophic lateral sclerosis"[Title/Abstract]) AND ((y_10[Filter]) AND (clinicaltrial[Filter] OR randomizedcontrolledtrial[Filter]))	304
Scopus	TITLE-ABS-KEY ( ( "Amyotrophic Lateral Sclerosis" OR "ALS") AND ( "pain" OR "pain management")) AND PUBYEAR > 2012 AND PUBYEAR < 2024 AND ( LIMIT-TO ( LANGUAGE, "English") OR LIMIT-TO ( LANGUAGE, "French") OR LIMIT-TO ( LANGUAGE, "Spanish") OR LIMIT-TO ( LANGUAGE, "Portuguese")) AND ( LIMIT-TO ( DOCTYPE, "ar"))	638
Clinicaltrial.gov	Amyotrophic Lateral Sclerosis\(ALS\ ) Pain management Study start date: 1 january 2013 to 23 november 2023	14
Cochrane-OVID	("Amyotrophic Lateral Sclerosis" OR "ALS") AND ("pain management")	6

### Eligibility criteria

Studies were considered eligible if these complied with all inclusion criteria: Completed randomized clinical trials (RCTs), open label RCTs and pilot trials with reported results, studies published in the last 10 years (2013–2023), studies written in English, Spanish, French and Portuguese, articles evaluating adult patients (> 18 years old) with a confirmed diagnosis of ALS, who have been treated with either pharmacological or non-pharmacological therapies for pain management, and studies with an objective evaluation of pain symptoms either as a primary or secondary outcome.

Reasons for exclusion were observational studies (case reports, case series, cohort, case–control, cross-sectional), commentaries, book chapters, scoping reviews, narrative reviews, posters and abstracts, studies with a combined population of children and adults with no differentiated results for each age group, studies written in a foreign language, studies with patients with no confirmed diagnosis of ALS, studies that do not evaluate pain as a variable, studies focused on pharmacological or non- pharmacological interventions for other symptoms in ALS different from pain.

#### Selection process

**Fig. 1:**
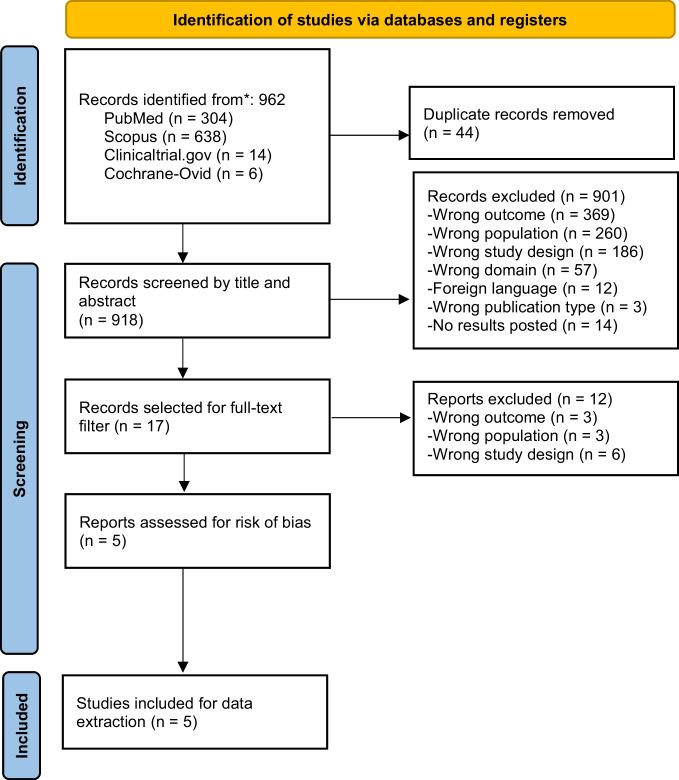
PRISMA flow diagram

The total number of articles were uploaded to the software system Rayyan after removal of duplicates. All authors were grouped in pairs. Each pair of authors screened an equal number of articles by title and abstract. Each author worked independently to screen their corresponding studies. To reduce biased decisions, a blind filter was applied, therefore decisions and labels of each reviewer were not visible to others. Disagreements between individual judgments were solved by a third author. After the title and abstract filter was applied, full-text screening was conducted by a different pair of authors, and disagreements were solved by a third. The remaining studies that complied with the inclusion criteria underwent risk of bias, validity, and quality assessment and were eligible for data extraction and further qualitative analysis **(**Fig. 1**).**

### Study risk of *bias* and quality assessment

**Fig. 2:**
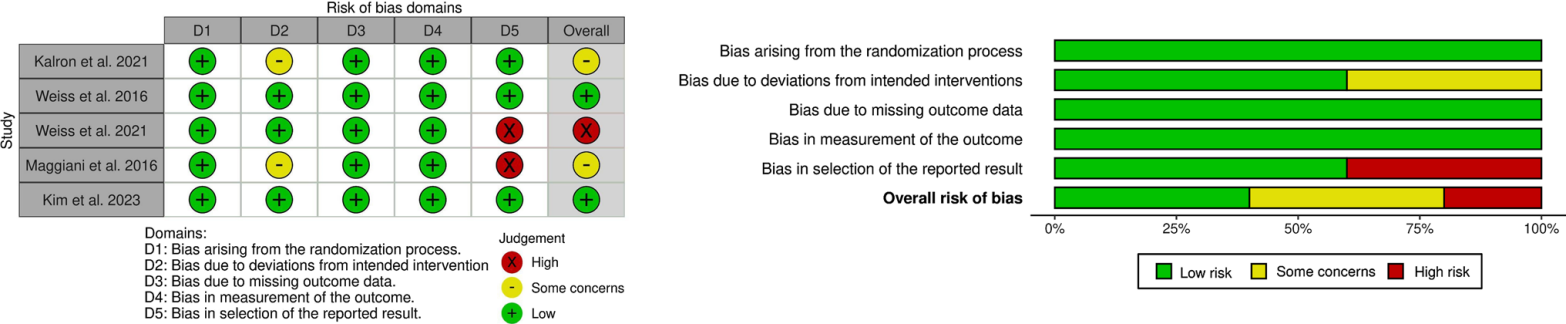
Risk of bias assessment summary. Summary of risk of bias assessment using Version 2 of the Cochrane Risk-of-Bias tool for randomized trials (RoB 2.0). Left: Risk of bias Traffic Light Plot, Right: Risk of Bias summary graph

RCTs were assessed for quality, validity, and risk of bias using the Version 2 of the Cochrane Risk-of-Bias tool for randomized trials (RoB 2) **(**Fig. 2**)**. Studies were distributed among pairs of authors, who worked independently. Any disagreements were solved by a third author. Additionally, to avoid reporting bias, clinical trials with no reported results or unpublished data were excluded from the review. Figures were elaborated with the risk-of-bias visualization tool (robvis) [46].

### Data collection process

Data collected from eligible studies was registered in an Excel format including both demographic and clinical data. Information from each study was collected by a reviewer and corroborated by another one. No automation tools were used for data extraction. The main outcome sought in each study was pain perceived by each patient according to the pain evaluation scale indicated in each study. Pain was taken into account if described as both, primary or secondary outcome. Other variables registered include participant characteristics (mean age, sex, ALS diagnostic criteria used, sample size) and intervention features (type, dosage, duration).

Given the limited number of included studies and heterogeneity among them, we opted for a descriptive characterization of such studies, for instance, no effect of measure was employed. Data was synthesized according to the type of intervention including pharmacological and non-pharmacological strategies.

## Results

This systematic review aimed to describe the efficacy of pharmacological and non-pharmacological therapeutic modalities for managing pain experienced by individuals diagnosed with ALS. Two previous systematic reviews published in the years 2013 [35] and 2017 [44] evaluated the efficacy of pharmacological therapy for alleviating pain in these patients and the side effects of the administered medications. The first one, conducted by Brettschneider et al. [35], included 886 articles of all types of RCTs and quasi-randomized controlled studies, published between 1980 and 2012, which used pharmacological treatment for pain, and excluded studies on treatment for cramps. The authors concluded that there was no evidence from RCTs for pain management in ALS, and that the effect of non-pharmacological treatments for controlling this symptom in ALS is unknown.

Similarly, Ng et al. [44] included 82 Cochrane systematic reviews in which they analyzed a total of nine reviews. These examined symptomatic treatment therapies for individuals with Motor Neuron Disease (MND) at both impairment and activity/participation levels. Such interventions targeted various symptoms including pain, cramps, spasticity, respiratory function support, sialorrhea, nutrition support, repetitive transcranial magnetic stimulation (rTMS), therapeutic exercise, and multidisciplinary care. Notably, none of the interventions specifically addressed pain management, leading to the conclusion that there is a lack of RCTs available for pain management in ALS patients.

Our study focused exclusively on the analysis of RCTs and one pilot trial regarding pharmacological and non-pharmacological treatment, published between 2013–2023. We initially found 962 articles, including 44 duplicates. Of the 918 evaluated by title and abstract, 901 were excluded because they reported outcomes other than pain; the study population was not diagnosed with ALS; the study design was not some type of RCT; the domain was unrelated to our research question; studies were written in languages other than English, Spanish, French or Portuguese and had no reported results. Seventeen articles were retrieved and read in full text, of which 12 were excluded because they evaluated patients with conditions other than ALS, had a cross-sectional study design, assessed pain in caregivers instead of patients, and had no objective evaluation of pain (Fig. 1).

Risk of bias, quality, and validity assessment was performed on these five articles [29, 43, 47–49] which were later selected for descriptive analysis. Risk of bias assessment was heterogeneous according to the RoB 2.0 criteria, two of the articles included in this study had a low risk of bias, two some concerns and one high risk. The domain analysis shows that all 5 (100%) studies included in our systematic review have low risk of bias arising from the randomization process, due to missing outcome data and measurement of the outcome. For domain 2 (bias due to deviations from intended intervention) two out of five studies evidenced some concerns and for domain 5 (bias in selection of the reported outcome) only one out of the five studies reported a high risk of bias **(**Fig. 2**).**

### Descriptive analysis

Table 2 shows the characteristics of the five studies analyzed in this systematic review. These included a total of 161 patients with a confirmed diagnosis of ALS, with a predominance of male participants (n = 104) and ages ranging from 35.8 to 80.2 years. The articles were published between 2016 and 2023, however, none of them reported pain as a primary study outcome. In four of the five studies, the diagnosis of ALS was established based on the El Escorial criteria. The duration of the interventions described ranged from 4 to 12 weeks.

**Table 2:** Characterization of included studies

Characteristics	Frequency
Location in which the study was conducted	
- Italy [43]	1
- Israel [47]	1
- USA [29, 48]	2
- Korea [49]	1
Age range (years)	
- 42.4—65.6 [43]	1
- 45.3 – 71.7 [47]	1
- 48 – 68 [29]	1
- 40.7 – 74.6 [48]	1
- 35.8—80.2 [49]	1
Sex (n = 131)	
- Male	104
- Female	57
Type of intervention	
- Pharmacological	3
- Non-pharmacological	2
Outcome	
- Primary	0
- Secondary	5
Duration of intervention	
- 4 weeks	1
- > 4 weeks	4
Evaluated outcome related to pain	
- Pain intensity	4
- Functionality	2
- Quality of Life	2
Professionals involved with the intervention	
- Physical therapist	1
- Exercise physiologist	1
- Osteopath	1
- Physician/Neurologist	2
Diagnostic criteria for ALS	4
- Revised El Escorial Criteria	
Severity score for ALS	
- ALS Functional Rating Scale (ALSFRS-R)	3

### Pharmacologic therapy

Weiss et al. [29, 48] conducted two phase 2 double-blind RCTs with Mexiletine. In 2016, these authors described the use of Mexiletine administered to 59 outpatients diagnosed with ALS, using a three-arm, randomized, double-blind, placebo-controlled design. Patients were randomized with a block randomization schedule in a 1:1:1 ratio, with 20 receiving Mexiletine at a dose of 900 mg/day divided into two doses; 300 mg/day divided into two doses; and placebo divided in two daily doses. For treatment allocation, participants were also randomized using a computer-generated permuted block randomization schedule, stratified by treatment allocation. Regarding the 900 mg dose, patients reported nausea as a side effect. With a 95% CI, a dose-dependent decrease in cramping and pain intensity was found. In the control group and in the group of patients receiving 300 mg Mexiletine there was no difference in pain intensity, whereas the administration of 900 mg reported significant differences compared to placebo (*p* = 0.005).

In 2021, using the same randomization method, Weiss et al. [48] reported the effects of oral Mexiletine in 20 patients. In this case, 8 patients received 300—600 mg/day, 6 patients 900 mg/day and 6 patients placebo. There was no difference in the intensity of pain experienced in the control group and the experimental group. However, they reported a decrease in cramping intensity (*p* = 0.044) with active Mexiletine treatment (300 and 600 mg combined) versus placebo from baseline to week 4, which may represent a decrease in pain as a secondary outcome. Due to the nausea reported in the first study [29], dosage was lowered from 900 to 600 mg per day in this study.

Kim et al. [49] in 2023 performed a multicenter, randomized, double-blinded, placebo-controlled, three-arm riluzole add-on clinical trial. It was conducted from August 2016 to September 2018 with a 12-week follow-up period. Patients were randomized to the 1.6 g or 2.4 g Mecasin or placebo group. Subjects were administered Mecasin and identical placebo tablets three times a day for a period of 12 weeks. For the low dose Mecasin group, a total of six tablets were administered, four of Mecasin (1.6 g) and two of placebo, whereas for the high dose Mecasin group, six tablets of Mecasin (2.4 g) were administered. In the placebo group, six tablets of placebo were administered. The primary endpoint was K-ALSFRS-R score changes between baseline and week 12. Secondary endpoints included changes in Visual Analog Scale for pain (VAS pain) scores, however, no significant differences were detected in VAS pain scores between groups (*p* = 0.916).

In synthesis, both pharmacological studies using Metilexine showed pain relief, in contrast, non-pharmacological interventions and the administration of Mecasin reported no change in pain relief.

### Non-pharmacological therapy

Maggiani et al. [43] conducted a pilot feasibility study to evaluate the effects of Osteopathic Manual Treatment (OMT) for ALS with pain, quality of life and goal attainment as secondary outcomes. In recognition of the frailty of ALS patients to manual therapies, these authors included 14 patients in the study. They used a single-blind design in which patients were randomly assigned (time: T0) using a randomly generated list to determine either conventional physiotherapy treatment or OMT.

Pain intensity was measured at three time points (baseline, T1 and T2) with the Brief Pain Inventory-Short Form, with a decrease in pain intensity between T1 and T2, but without significant differences. However, pain severity decreased between T1 and T2 (PSI, pain severity index of the BPI, *p* = 0.05).

Kalron et al. [47] conducted a longitudinal parallel-group RCT, with 32 outpatients (of whom only 28 completed the study), who were randomly assigned in a 1:1 ratio to either the combined intervention group or the control (stretching) group. The intervention was delivered by two health professionals (physiotherapist and physiologist). Adherence to exercise was monitored through a self-report diary and telephone contact every two weeks.

Authors assessed pain intensity as a secondary outcome, measured by a subscale of the SF36. The intervention consisted of 12 weeks of outpatient aerobic and strength training, with three measurements: baseline, 6 weeks and 12 weeks. No significant differences in pain experienced in the different groups were reported. Study characteristics are summarized below **(**Tables 3 and 4).

**Table 3:** Summary of data extraction—sociodemographic variables

Author	Year	Study design	Aim	Total sample size (n)	Placebo sample size (n)	Therapy sample size (n)	Age (SD) years	male/ female (n)
[43]	2016	Feasibility Pilot Study	Assess safety, feasibility, tolerability and satisfaction of OMT in a preliminary series of ALS outpatients. (p. 60)	14	FKT T0 7	OMT T0 7 OMT T1 10	OMT T0 54.0 (11.6) FKT T0 51.0 (6.5) OMT T1 50.3 (7.9)	OMT T0 5/2 FKT T0 5/2 OMT T1 8/2
[47]	2021	RCT	Compare the effectiveness of a combined aerobic, strength, and flexibility training program compared with flexibility training alone on disease-specific and generic health-related symptoms in ambulatory ALS patients (p. 1858)	28	14	14	58.5 (13.2)	17/11
[29]	2016	Phase 2 double blind RCT	Determine the safety and tolerability of mexiletine in patients with sporadic amyotrophic lateral sclerosis (p. 1474)	59	20	Mx 900 mg (19) Mx 300 mg (20)	Mx 900 mg: 58.0 (10.0) Mx 300 mg 59.2 (7.1) Placebo: 57.0 (7.0)	Mx 300 mg: 14/6 Mx 900 mg: 12/7 Placebo: 10/10
[48]	2021	Phase 2 double-blind RCT	Determine Mexiletine effects on pharmacodynamic markers of cortical and axonal excitability (p. 3)	20	6	Mx 300 mg (8) Mx 600 mg (6)	Mx 300 mg: 58.5 (10.8) Mx 600 mg: 60.5 (14.1) Placebo: 52 (11.3)	Mx 300 mg: 5/3 Mx 600 mg: 4/2 Placebo: 5/1
[49]	2023	Phase 2a multicenter double blinded RCT	Evaluate the efficacy and safety of Mecasin in patients with ALS (p.2)	30	10	Mc 1.6 g (10) Mc 2.4 g (10)	Mc 1.6 g: 54,9 (12,3) Mc 2.4 g: 58,7 (6,3) Placebo: 57,1 (10,0)	Mc 1.6 g: 5/5 Mc 2.4 g: 6/4 Placebo 8/2

Note: Randomized Controlled Trial (RCT), Osteopathic Manual Treatment (OMT), Metilexine (Mx), Mecasin (Mc), Amyotrophic Lateral Sclerosis (ALS), Usual care physiotherapy (FKT)

**Table 4:** Summary of data extraction—therapeutic variables

Type of intervention	Therapy	Dosage/ frequency	Duration intervention	Pain evaluation scales/Type of pain explored	Results	Overall effect in pain relief
Non- Pharmacologic [43]	Osteopathic manual treatment (OMT)	1x/week for the first 4 weeks, and fortnightly for 8 weeks	12 weeks	Brief Pain Inventory (BPI) Type of pain nonspecific	**-**T0: 57% (8 out of 14) reported no pain **-**T1: 43% (6 out of 14) reported no pain **-**T2: 50% (7 out of 14) reported no pain -No significant differences were found in pain intensity between the OMT and FKT groups in the T0-T1 and T1-T2 periods **-**T0**-**T1: no significant differences were found in pain intensity change scores between the OMT and FKT groups **-**T1**-**T2: a trend towards pain reduction was observed for the OMT group (*p* = 0.05)	No change in pain
Non- Pharmacologic [47]	Combined Aerobic and Strength Program: Aerobic Training (20–30 min) + Recumbent cycling at 40–60% of the heart rate reserve + Flexibility (10 min) + Strength Training (20 min)	2 sessions per week, 50–60 min per session	24 sessions over 12 weeks	Nonspecific Type of pain nonspecific	-No statistically significant changes in pain over time were observed in both cases and controls (p > 0.05, F for the time factor) -The interaction between time and the intervention group did not reach statistical significance (p > 0.05, F for the time x group factor)	No change in pain
Pharmacologic [29]	Mexiletine	300 and 900 mg/d	12 weeks	Visual Analog Scale (VAS pain) and Muscle cramp diary Type of pain muscle cramp pain intensity	-Significant reduction in muscle cramps: 31% with 300 mg, 16% with 900 mg -Decreased intensity in cramp-associated pain: 45% with 300 mg and 25% with 900 mg -Mexiletine is safe at 300 and 900 mg, however, higher doses (900 mg/d) reported more adverse effects. Mexiletine decreases in a dose-dependent way frequency and severity of muscle cramps	Relief
Pharmacologic [48]	Mexiletine	300 and 600 mg/d	4 weeks	Visual Analog Scale (VAS pain) Type of pain muscle cramp pain intensity	- Cramp intensity was 1.3 units lower than placebo at weeks 3 and 4 for subjects on both 300 and 600 mg/day (*p* = 0.044) -Cramp frequency was not significant among all subjects and those who reported at least 10 or more cramps during the 30 days prior to baseline - At 600 mg/day there was a relative reduction of 56% among all subjects and 47% among those with more than 10 cramps at baseline -There was no correlation between muscle cramp frequency (mean per week) or duration of fasciculations (percentage of days) and axonal excitability parameters	Relief
Pharmacologic [49]	Mecasin tablets-composed of 9 different herbs (Curcuma longa 0.48 g/tablet, Salvia miltiorrhiza 0.48 g/tablet, Gatrodia elata 0.48 g/tablet, Pseudocydonia sinensis 0.24 g/tablet, Paeonia lactiflora 0.24 g/tablet, Polygala tenuifolia 0.24 g/tablet, Glycyrrhiza uralensis 0.24 g/tablet, Atractylodes japonica 0.24 g/tablet, Aconitum carmichaeli 0.12 g/tablet)	1.6 g and 2.4 g thrice daily	12 weeks	Visual Analog Scale (VAS pain) Type of pain nonspecific	Pain was evaluated as a secondary outcome. No significant differences were identified in VAS pain (*p* = 0,916). Incidence of adverse effects was similar in placebo and Mecasin groups	No change in pain

## Discussion

Despite being present in more than 70% of patients [50], pain in ALS has long been underestimated and inadequately addressed within the medical community [2]. One of the key contributors to the sub estimation of pain in ALS lies in the inherent communication barriers faced by patients [51]. The progressive loss of motor function hampers the ability to self-report pain adequately, creating a silent struggle that frequently goes unnoticed [51]. Medical professionals, understandably focused on motor symptoms, more frequently associated with ALS, may inadvertently sideline the nuanced and intricate dimension of pain experienced by these individuals [52].

Our results provide insight into what is not effective in relieving pain in patients with ALS. Evidence from studies on osteopathy and combined aerobic and strength programs show that these therapies do not significantly alleviate pain in ALS patients [47]. However, it is noteworthy that the combined aerobic and strength programs have demonstrated improvements in other aspects, such as respiratory function, mobility, and overall well-being, particularly in ambulatory ALS patients [47]. Similarly, Mecasin in both, low and high doses, does not alleviate pain in ALS patients, as evidenced by the lack of significant differences in VAS pain scores, although it may delay symptomatic progression without major adverse effects [49].

Contrastingly, Mexiletine is the only pharmacological therapy that has demonstrated a significant reduction in pain, reducing the frequency and intensity of cramps. This shows promise as an effective option for pain management in ALS patients, however, further clinical trials are necessary before recommending Mexiletine for pain management. It is essential to remain vigilant in monitoring such symptoms that may not pose an immediate threat to a patient's life, but significantly impact their quality of life [53].

The EAN guidelines recommend addressing pain through both pharmacological and non-pharmacological interventions. Pharmacological options include opioids for managing symptoms of breathlessness, and benzodiazepines for anxiety-exacerbated breathlessness, which indirectly alleviate pain [54]. Non-pharmacological approaches such as tailored exercise programs can also help manage muscle pain by maintaining joint range of movement and preventing contractures [54].

Lack of efficacy of pain management strategies could be attributed to a decline in reported pain symptoms in ALS patients. To exemplify, a clinical trial of 55 patients diagnosed with ALS, using psychological and cognitive tests to measure patients' interoception sensitivity, found that ALS patients have significantly lower sensory interoception. Such decreased perception of pain in these patients can explain the lack of this symptom´s reports by patients and their caregivers [55]. In addition, some studies suggest that it may be due to a degenerative process involving the insula, but further studies correlating insular degeneration and sensory interoception deficits in ALS are needed [55, 56].

Besides a decline in reported pain symptoms, lack of efficacy of pain management therapies could also be attributed to the heterogeneity of underlying pain mechanisms reported by patients with ALS. To exemplify, musculoskeletal pain usually develops at the later stages of the disease and is frequently related to chronic muscle wasting, muscle atrophy and decreased muscle tone [52]. Contrastingly, muscle cramps often occur at the earlier stages of the disease due to motor neuron hyperexcitability which leads to pain from uncomfortable postures and abnormal ambulation [57]. In addition, irregular proprioceptive inputs in the spinal cord can explain spasticity, eventually leading to muscle fatigue and muscle cramps referred to as painful symptoms [52]. Taking into account the variable mechanisms of pain that occur in ALS, an individualized therapeutic approach emerges as a potential strategy for symptom control.

Furthermore, the EAN guidelines emphasize the importance of managing depression and anxiety in ALS patients. These mental health issues can significantly affect patients' quality of life and exacerbate their perception of pain. Identifying and treating underlying causes of anxiety, such as breathing difficulties, fear of death, pain, and loss of functionality, is crucial. The guidelines recommend psychological support and pharmacological interventions, such as short-acting anxiolytics and SSRIs, to manage these symptoms [54]. In advanced or late-stage ALS, where psychotherapy is not feasible, pharmacotherapy is advised as the first-line treatment [54].

These findings emphasize the need for awareness regarding the overall welfare of our patients. Many therapies primarily target the physical impairments associated with this condition, often relegating pain and other symptoms to secondary consideration. While numerous studies focus on the positive outcomes of physical therapies, such as improved eating without broncho-aspiration and enhanced breathing [41], the overall well-being of patients, who frequently experience pain without a specific treatment, is equally important.

The EAN guidelines strongly advocate for a multidisciplinary approach to the management of ALS, recognizing that effective care involves addressing both motor and non-motor symptoms. A multidisciplinary team (MDT) should include various healthcare professionals to assess, manage, and review comprehensive aspects of the patient's health, such as nutritional status, muscle problems, respiratory function, and psychological needs [54]. This approach ensures that all facets of the patient's well-being are addressed, enhancing their quality of life significantly.

As mentioned earlier, RCTs focusing on pain management in ALS patients are limited. However, it is worth noting other types of studies that have been conducted in the pursuit of an optimal pain management for these patients. In a case report by Koda et al. [58] in 2021, Chinese Scalp Acupuncture and auricular acupuncture were applied to an ALS patient, evidencing pain relief and enhancement in quality of life. Similarly, a nationwide online survey for ALS patients carried out in France in 2022, reported benefits in motor and non-motor symptoms, including pain, with the use of cannabidiol oil and cannabis weed [54]. Bialkowska et al. [59] evaluated the effectiveness of concomitant neurofeedback in rehabilitation therapy in a 71-year-old patient with ALS following subdural stem cell implantation, evidencing a decrease in the VAS score by 6 points. Such promising results in previous studies could contribute to a future evaluation of the benefits of these therapies in RCTs.

Numerous questions have been raised by this systematic review, notably regarding the scarcity of RCTs that focus on pain measurement in ALS patients. Our comprehensive examination of the available data has revealed several intriguing findings. Physicians prioritize preventing the premature death of these patients, focusing primarily on the respiratory and stomatognathic system, as has been discussed throughout this article. Rhiannon et al. [53], conducted a study involving 636 individuals with ALS, of which 69% reported experiencing pain, predominantly of mild intensity. They concluded that pain significantly impacts quality of life, underscoring its importance as a symptom. Furthermore, the study highlighted a positive correlation between pain and depression, indicating that higher pain intensities could exacerbate depression severity. This suggests a complex interplay that detrimentally affects patients' experiences with the disease, underscoring the need for further research to unravel these connections.

In order to improve quality of life in patients with ALS, we recommend a multidisciplinary therapy, with a comprehensive psychological support, that acknowledges both motor and non-motor symptoms, in addition to pharmacological and non-pharmacological strategies for symptom control. Furthermore, we recommend the development of RCTs that evaluate pharmacological and non-pharmacological interventions for pain in ALS as a primary outcome.

In terms of limitations, four out of five studies [29, 43, 48, 49] included in this review reported a small sample size, for which extrapolation of results and clinical recommendations must be done with caution. Overall, the main limitation of this systematic review is the small number of RCTs included which difficulted the quantitative analysis and statistical significance of our results. Additionally, given that pain was evaluated and reported only as a secondary outcome, there is a higher risk of reporting bias which can also limit this study.

## Conclusion

Clinical trials focusing on pain management strategies for ALS patients are limited and are frequently assessed as a secondary outcome. Among the available pharmacological therapies, Mexiletine evidenced positive effects for pain relief. Contrastingly, non-pharmacological therapeutic and Mecasin high and low doses options reported no change in pain. Medical professionals, understandably focused on immediate life-threatening aspects, may inadvertently sideline the nuanced and intricate dimension of pain experienced by patients with ALS.

## Supplementary Information

Below is the link to the electronic supplementary material.

Supplementary file1 (DOCX 32 KB)

## Data Availability

Data will be made available upon request.
